# Cadmium and copper reduce hematopoietic potential in common carp (*Cyprinus carpio* L.) head kidney

**DOI:** 10.1007/s10695-012-9738-6

**Published:** 2012-10-20

**Authors:** Elzbieta Kondera, Malgorzata Witeska

**Affiliations:** Department of Animal Physiology, University of Natural Sciences and Humanities, Prusa 12, 08-110 Siedlce, Poland

**Keywords:** Carp, Hematopoiesis, Fish, Proliferation, Apoptosis, PCNA, Caspase 3

## Abstract

The effects of cadmium and copper on activity of common carp head kidney hematopoietic tissue were evaluated. The fish were subjected to short-term (3 h, Cd-s and Cu-s) or long-term (4 weeks, Cd-l and Cu-l) exposures to 100 % 96hLC_50_ or 10 % 96hLC_50_, respectively. Head kidneys were isolated weekly from 5 fish of each group for 4 weeks (post-short-term exposure and during long-term exposure). Percentage of early blast cells among the hematopoietic precursors was calculated. Proliferative and apoptotic activity were evaluated using immunocytochemical staining for proliferating cell nuclear antigen (PCNA) and caspase 3, respectively. Hematopoietic activity was calculated as the ratio of proliferating to apoptotic cells. All metal exposures induced an increase in frequency of early blast cells. The frequency of proliferating (PCNA-positive) cells also significantly increased. A considerable and significant increase in the frequency of apoptotic cells was the most pronounced effect of metal exposures. Both short-term and long-term treatments caused similar effects, but in case of Cd exposures, the reaction was more pronounced. All metal exposures reduced hematopoietic potential of fish measured as the ratio of proliferating to apoptotic precursor cell frequency. However, in all cases, hematopoietic activity was higher than 1 showing that the rate of repair of hematopoietic tissue prevailed over destruction.

## Introduction

In most teleost fishes head kidney, *pronephros* plays an important role as main hematopoietic organ and blood cell reservoir (Fange 1994; Houston et al. 1996; Fijan 2002a, b; Romano et al. 2002; Moritomo et al. 2004; Rombout et al. 2005; Gangopadhyay and Homechaudhuri 2011). According to Wendelaar Bonga (1997) and Weyts et al. (1999), *pronephros* shows not only hematopoietic activity but also is a lymphoid and endocrine organ. However, head kidney is not the only site of hematopoiesis in fish. According to various authors (Glomski et al. 1992; Houston et al. 1996; Kobayashi et al. 2006, 2007, 2008; Zapata et al. 2006; Mulero et al. 2007 and Santos et al. 2011), other organs such as spleen, gut-associated lymphoid tissue (GALT), mucosa-associated lymphoid tissue (MALT), and intertubular tissue of trunk kidney (*mesonephros*) may also show hematopoietic activity. In some fish species, several hematopoietic organs are active, while in the others only one of them (Liu et al. 2004; Mulero et al. 2007; Patel et al. 2009).

Morphology of hematopoietic tissue in fish is quite well known (e.g., Fange 1994; 2002a, b), but little information is available on the effects of environmental factors on its structure and activity, and studies of hematopoietic tissue are seldom included in evaluation of physiological effects of toxic agents on fish. Hematopoietic activity of head kidney tissue involves proliferation of stem cells and early precursors of all cell lines, differentiation and maturation, as well as apoptosis, and the rate of all these processes is a key factor that determines efficiency of hematopoiesis. Stem cell renewal and precursor cell proliferation are counterbalanced by apoptosis in functionally inactive or terminally differentiated cells (McKenna and Cotter 1997). Apoptosis plays an important role in regulating early progenitor and stem cells, and particularly for the development and function of lymphoid cells (Domen 2000). Cell proliferation involves the presence of PCNA—proliferating cell nuclear antigen—a protein found only in cells undergoing mitosis. The marker protein of apoptosis is caspase 3—an enzyme participating in degradation of nuclear and cytoplasmic proteins during this process. This caspase is commonly defined as effector caspase (Migliarini et al. 2005). Evaluation of both precursor cell proliferation and apoptosis rate is applied in hematological studies to evaluate the rate of cell turnover and hematopoietic activity (Thiele et al. 1997; Kvasnicka et al. 1999). The presence of PCNA in fish hematopoietic tissue was confirmed by Leung et al. (2005) for *Danio rerio*. Both fish PCNA and caspase 3 have been proved to react with mammalian (mouse or rabbit) monoclonal antibodies, and these antibodies were successfully used for evaluation of proliferation and apoptosis of various cells in *Thalassoma pavo* (Monteiro et al. 2009), *Oreochromis niloticus* (Brunelli et al. 2011), and *Salmo salar* (Yousaf et al. 2012).

Cadmium and copper are well known to induce hematotoxicity in fish, often resulting in anemia and immunosuppression (e.g., Svobodova et al. 1994; Dethloff et al. 1999; Vosyliene 1999; Joshi et al. 2002; Drastichova et al. 2004; Seong-Gil et al. 2004; Ates et al. 2008; Witeska et al. 2009, 2010). Sometimes the values of hematological parameters of intoxicated fish fluctuate, and their changes are not always directly related to metal concentrations and time of exposure or time post-exposure (Ruparelia et al. 1990; Shah and Altindag 2005; Witeska et al. 2010). These fluctuations may result from translocation of cadmium and copper within the organism, and their toxic action on various functions at different time. Cadmium and copper probably affect not only circulating blood cells, but also newly developing ones in hematopoietic tissue. Very scarce data concerning hematopoietic effects of heavy metals in fish (Garofano and Hirshfield 1982; Ghosh et al. 2007; Som et al. 2009) and mammals (Lutton et al. 1984; Mitsumori et al. 1998; Van Den Heuvel et al. 1999; 2001; Celik et al. 2005, 2009) indicate that they are cytotoxic to precursor cells, and various cell lineages show different sensitivity to metal toxicity.

The aim of present study was to evaluate the effects of copper and cadmium under various exposure conditions (concentration and time) on hematopoietic potential of common carp head kidney.

## Materials and methods

Common carp (*Cyprinus carpio* L.) 6-month-old juveniles of body mass 21.5 ± 8.3 g were harvested in October from the rearing ponds of the Inland Fisheries Institute in Żabieniec and brought to the Department of Animal Physiology, University of Natural Sciences and Humanities in Siedlce in plastic bags with water and supplied with pure oxygen. Before the experiment, the fish were acclimated for 4 weeks to the laboratory conditions in the flow-through tank, at 17–18 °C. Water was constantly aerated, and O_2_ concentration was 6.1–8.0 mg/dm^3^ of O_2_ (66–87 % saturation), while concentration of NO_2_^−^ 0.02–0.06 mg/dm^3^, and NH_4_^+^ 4.6–7.1 mg/dm^3^. The fish were fed Aller Aqua Classic 4 mm feed at the rate of 2 % of stock mass per day. Prior to the experiment survival tests were performed, and 96hLC_50_ values were calculated using the probit method for both metals. Fish were exposed for 3 h to Cd and Cu concentrations equal to 100 % of 96hLC_50_ (6.50 and 0.75 mg/dm^3^ for Cd and Cu, respectively)—groups Cd-s and Cu-s or for 4 weeks to 10 % of 96hLC_50_ (0.65 and 0.075 mg/dm^3^, respectively)—groups Cd-l and Cu-l. Control group (C) was kept in clean tap water (<0.3–1 μg/dm^3^ of Cd, 2–33 μg/dm^3^ of Cu). Experimental solutions were made using CdCl_2_ × 2½ H_2_O and CuSO_4_ × 5H_2_O, and 3/4 was renewed everyday without disturbing fish. The fish were kept in 100 dm^3^ aerated tanks (10 fish in each), and fed Aller Aqua Classic 4 mm (1 % of stock mass, once a day before water renewal). Five fish were sampled weekly for 4 weeks from each experimental group and killed for head kidney isolation. Water quality parameters were measured everyday (Table 1) using portable DO meter (HANNA instruments HI 9143), pH meter (ELWRO PRL TN 5123) ,and kits for nitrogen metabolites (Visocolor^®^ ammonium 0,2–10 mg/dm^3^ and Visocolor^®^ nitrite 0,05–2 mg/dm^3^, Machery Nagel). Water hardness values were provided by the water supplier (www.pwik.siedlce.pl).

**Table 1:** Water quality parameters during the experiment

Parameter	Group		
	C	Cu-s + Cu-l	Cd-s + Cd-l
Temperature (°C)	17.0 ± 0.5	17.1 ± 0.5	17.2 ± 0.6
Hardness (mg/dm^3^ CaCO_3_)	179–198		
pH	6.8–7.0		
O_2_ (mg/dm^3^)	8.9 ± 0.6	9.0 ± 0.2	8.8 ± 0.5
NO_2_^−^ (mg/dm^3^)	0.03 ± 0.01	0.06 ± 0.02	0.04 ± 0.02
NH_4_^+^ (mg/dm^3^)	3.9 ± 0.6	5.2 ± 0.8	6.1 ± 1.6

Five fish were sampled weekly for 4 weeks from each experimental group and killed for head kidney isolation. The kidneys were sliced and smeared on degreased slides and dried at room temperature for 24 h. Two preparations from each fish were stained Giemsa and May-Grunwald for cytological analysis. Hematopoietic precursor cells were identified (22 types of cells, according to Fijan 2002a, b, and Kondera 2011). Percentage of early blast cells was calculated per 500 cells in each preparation.

Immunocytochemical staining was also performed to identify proliferating cells (showing PCNA), and cells undergoing apoptosis (showing caspase 3) using monoclonal mouse antibodies anti-proliferating cell nuclear antigen clone PC 10 (Dako Cytomation) selectively binding to the PCNA antigen, and anti-Caspase 3 (active) antibody produced in rabbit, (Sigma) binding to active caspase 3. Dried preparations were hydrated with deionized water, and then peroxidase activity was blocked using 3 % H_2_O_2_ (Trace Pur, Merck). The antibody solution diluted 1:300 was applied and left on slides for 1 h at room temperature. Cells with antibody–protein complexes were visualized using Dako Cytomation En Vision + System-HRP for use with mouse primary antibodies, and Dako Cytomation En Vision + System-HRP for use with rabbit primary antibodies, respectively, according to the producer’s instruction. The PCNA-positive and caspase 3-positive cells stained brownish and were easily distinguishable from other cells (stained light blue with hematoxylin). Negative control staining was also performed (the preparations were treated the same way except for incubation with antibodies) and resulted in no color reaction. The cells were counted in at least 6 fields, and percentage of proliferating and apoptotic cells was calculated per 300 cells in each preparation.

All preparations were preserved with Histokitt, Glaswarenfabrik Karl Hecht GmbH Germany, covered with cover glass, and viewed using Nikon Eclipse E600 microscope at 1,000× magnification. Photographs were done using Nikon—Eclipse E600 microscope connected with digital Nikon Coolpix camera and computer image analysis system CoolView (Precoptic, Poland).

The results were subjected to statistical evaluation of significance of differences in values of all parameters between the control and metal-exposed groups using Mann–Whitney U test (*Statistica* 9.0) at *p* ≤ 0.05.

The study obtained agreement of the III Local Ethical Committee at the Warsaw University of Life Sciences (No 41/2008).

## Results

**Fig. 1:**
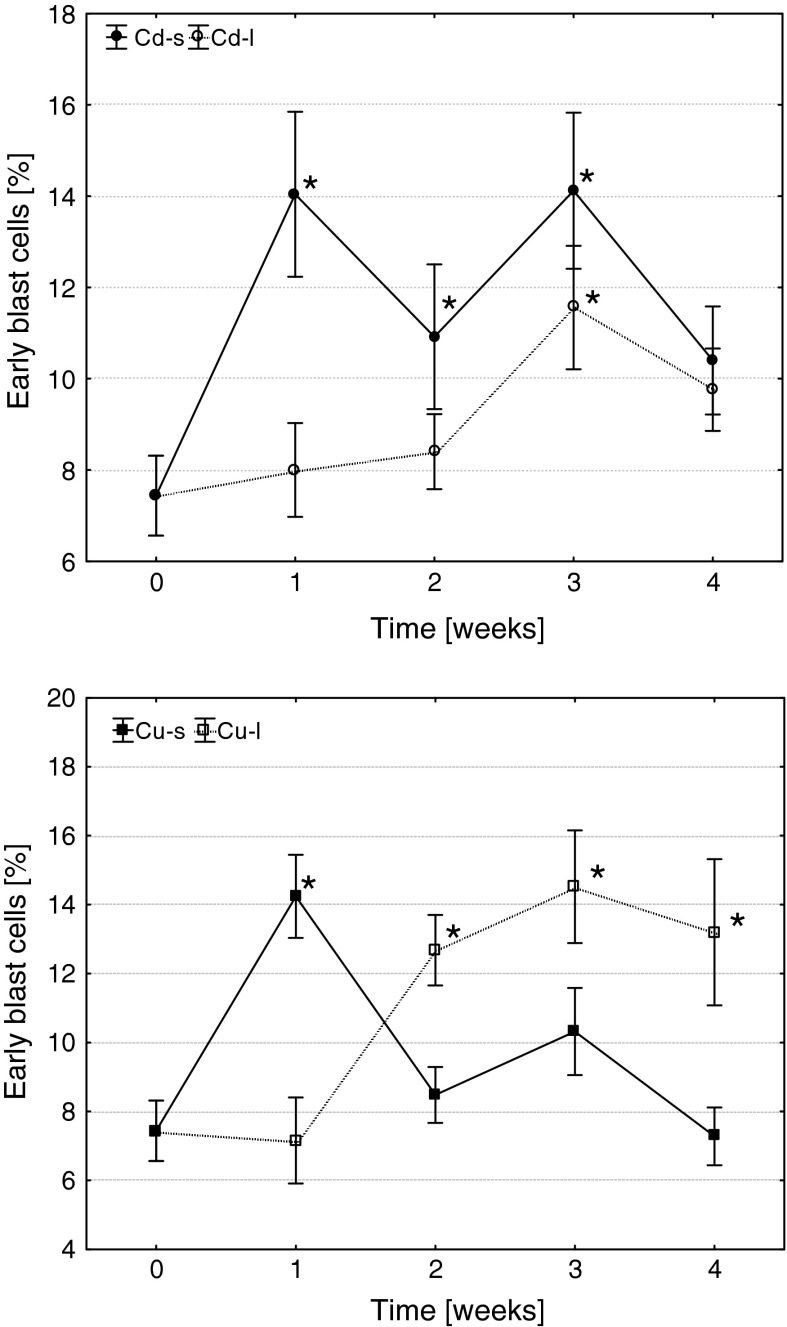
The effects of Cd and Cu on the frequency of early blast cells in head kidney hematopoietic tissue of common carp (arithmetic mean ± s.e., *different from the value before metal exposure (time 0), *p* ≤ 0.05, U Mann–Whitney test). Cd-s, Cu-s and Cd-l, Cu-l—groups subjected to short- or long-term exposures, respectively

**Fig. 2:**
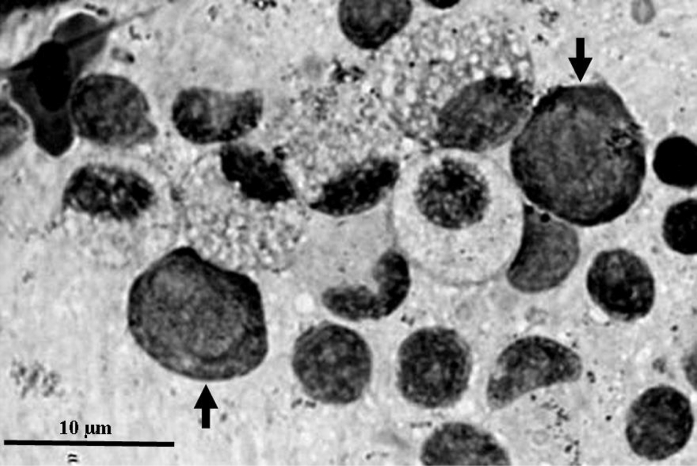
Early blast cells

Both metals induced significant changes in frequency of blast cells, as well as proliferating and apoptotic precursors. Short-term exposures to Cd and Cu resulted in an increase in the frequency of early blast cells in head kidney of carp in 1 week after the treatments (Figs. 1, 2). The level of blasts in Cd-s fish remained significantly elevated until the 3-week post-exposure, while in Cu-s group decreased already in the 2 weeks. On the contrary, during long-term exposure, Cd induced only transient increase in blast frequency (3 weeks), while the fish from Cu-l group showed significantly elevated level of early blast cells beginning from the 2 weeks of exposure until the end of the experiment.

**Fig. 3:**
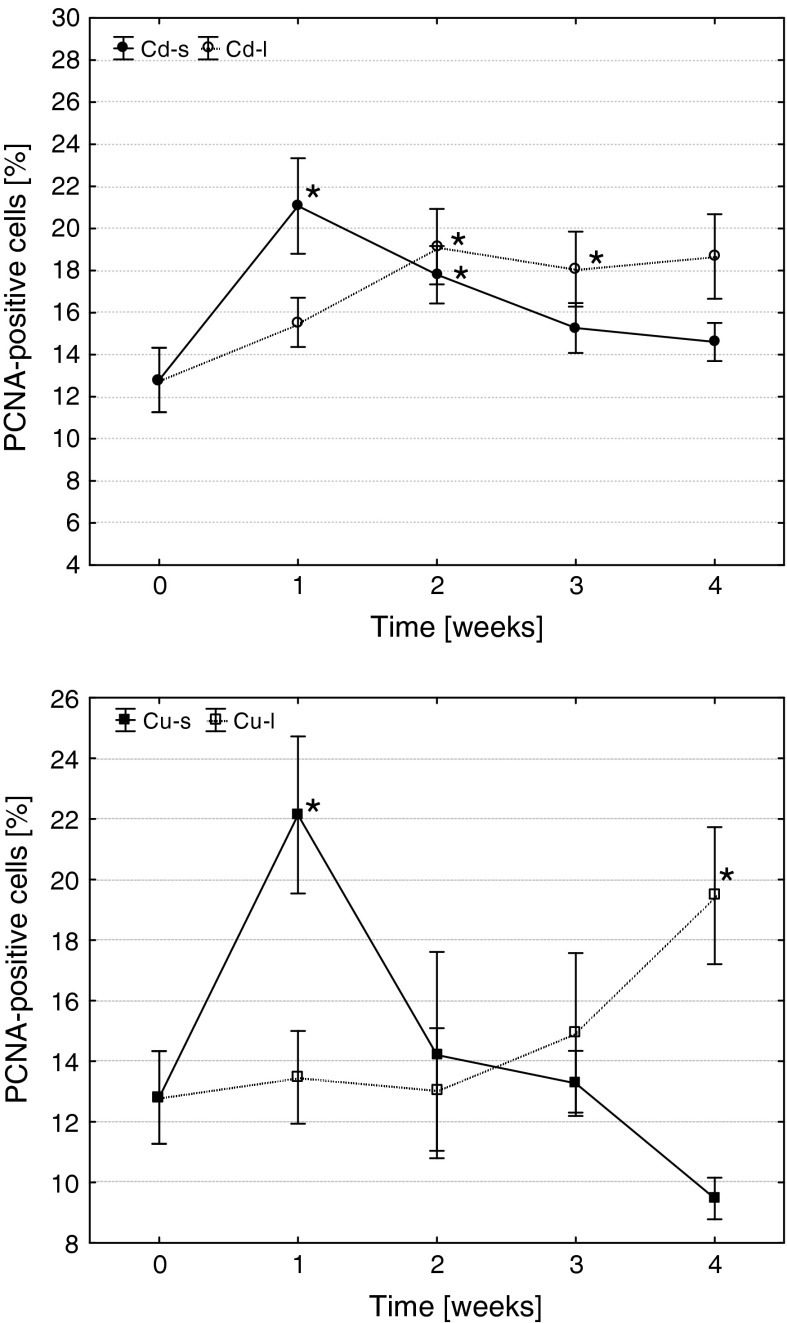
The effects of Cd and Cu on the frequency of proliferating cells in common carp head kidney hematopoietic tissue (arithmetic mean ± s.e., *different from the value before metal exposure (time 0), *p* ≤ 0.05, U Mann–Whitney test). Cd-s, Cu-s and Cd-l, Cu-l—groups subjected to short- or long-term exposures, respectively

**Fig. 4:**
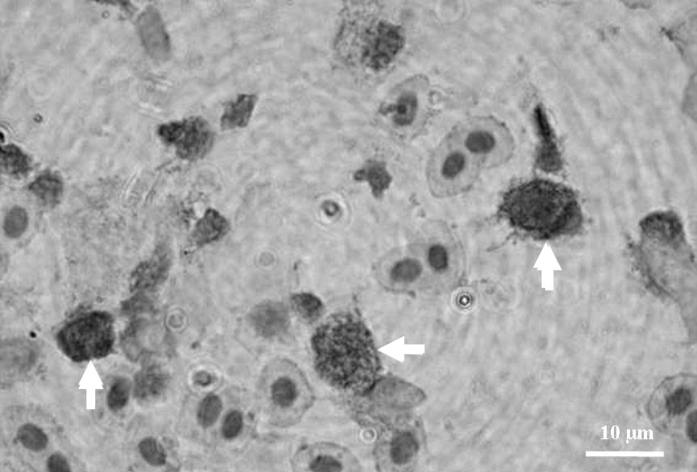
PCNA-positive cells

Similarly, also the frequency of proliferating (PCNA-positive) cells significantly increased in 1 week after short-term exposures (Fig. 3, 4). In Cd-s group, the level of cells undergoing mitosis remained elevated until the 2-week post-exposure, while in Cu-s proliferation rate quickly decreased to the control level. During long-term Cd exposure, a significant increase in precursor cell proliferation took place in 2 weeks, and their level remained elevated until the end of the experiment. The reaction of fish from Cu-l group was different: a significant increase in frequency of proliferating cells took place at the end of the experiment (in 4 weeks of exposure).

**Fig. 5:**
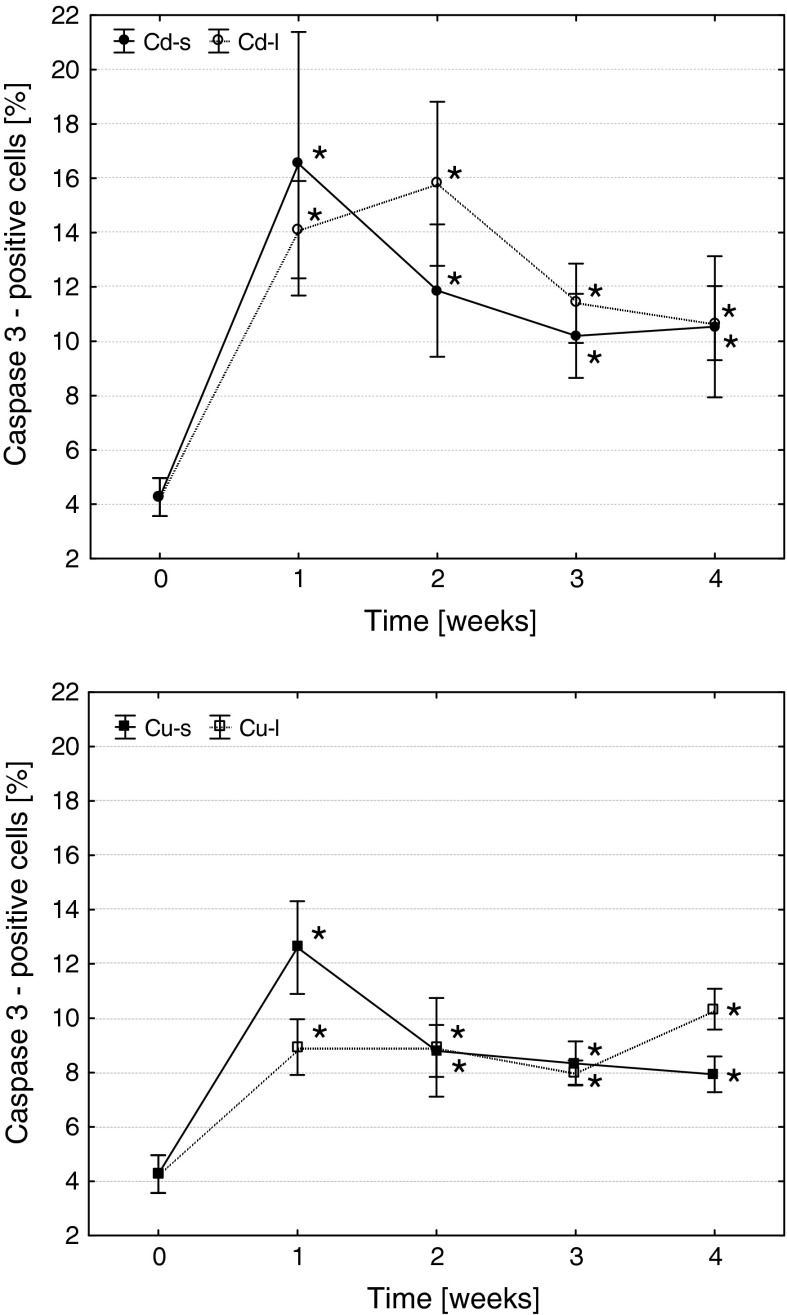
The effects of Cd and Cu on the frequency of apoptotic cells in common carp head kidney hematopoietic tissue (arithmetic mean ± s.e., *different from the value before metal exposure (time 0), *p* ≤ 0.05, U Mann–Whitney test). Cd-s, Cu-s and Cd-l, Cu-l—groups subjected to short- or long-term exposures, respectively

**Fig. 6:**
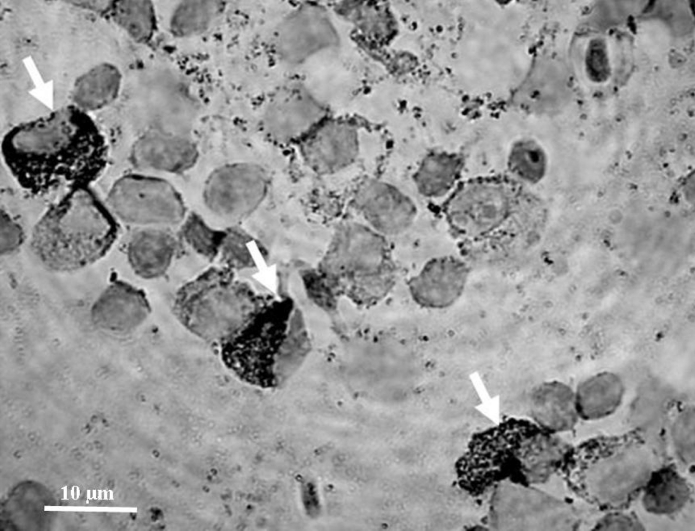
Caspase 3-positive cells

Both metals induced a significant increase in the frequency of apoptosis of hematopoietic precursor cells (Fig. 5, 6). Both short-term and long-term exposures caused similar effects, but in case of Cd exposures, the reaction was more pronounced.

**Fig. 7:**
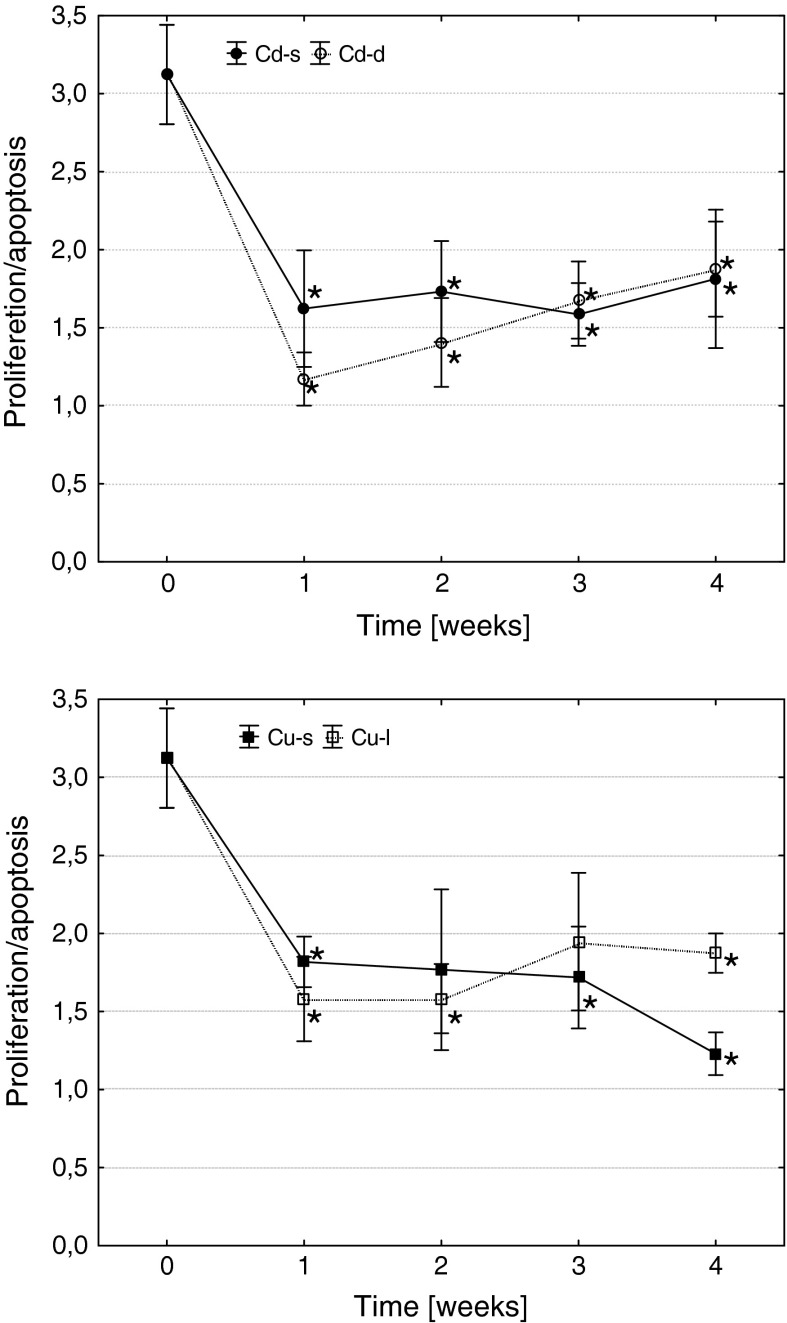
The effects of Cd and Cu on cell turnover rate in common carp head kidney hematopoietic tissue (arithmetic mean ± s.e., *different from the value before metal exposure (time 0), *p* ≤ 0.05, U Mann–Whitney test). Cd-s, Cu-s and Cd-l, Cu-l—groups subjected to short- or long-term exposures, respectively

These changes resulted in a reduction of hematopoietic potential in all metal-exposed fish, measured as the ratio of frequencies of precursor cell proliferation and apoptosis (Fig. 7). It is noteworthy that in case of short-term copper exposure, reduction in hematopoietic activity at the end of the experiment was deeper than in group subjected to short-term cadmium intoxication or to fish subjected to a long-term copper treatment. However, in all cases, hematopoietic activity was over than 1 showing that the frequency of proliferating cells was all the higher than percentage of precursors undergoing apoptosis.

## Discussion

The obtained results show that cadmium and copper disturbed hematopoiesis in carp but on the other hand indicate a considerable compensatory potential of carp hematopoietic system. The pattern of changes after short-term exposures (a rapid increase in cell proliferation rate and early blast frequency, accompanied by an increase in apoptotic rate) and during long-term treatments (gradual increase in the values of these parameters during the exposures) was different but the final effect—reduction in cell turnover rate was very similar. The increase in apoptotic rate was higher when compared to acceleration of precursor cell proliferation. No anemia was observed in peripheral blood or a significant reduction in leukocyte count, and the most pronounced effect of metal exposures was significantly reduced frequency of peripheral phagocytes (neutrophils and monocytes), accompanied by reduction in their metabolic activity (Kondera and Witeska 2012, and unpublished data of the same study). These results indicate that hematopoietic potential of carp head kidney tissue was reduced due to the increase in apoptotic cell destruction since no necrosis or other visible cell damage was observed.

Hematological effects of heavy metal intoxication of fish were extensively studied, but very little data concerning metal-induced alterations in hematopoietic system are available. According to Garofano and Hirshfield (1982), 2-h exposure of *Ictalurus nebulosus* at 61.3 mg/dm^3^ of Cd resulted in complete destruction and elimination of all hematopoietic precursor cells in head kidney hematopoietic tissue over 24-h post-exposure, and the only cells present after intoxication were mature erythrocytes. Also, Saxena et al. (1992) mentioned damage of *Heteropneustis fossilis* hematopoietic tissue caused by this metal. Considerable hematopoietic disturbances were also observed in *Labeo rohita* subjected to Cu treatment: 50 % 72hLC_50_—25 mg/dm^3^ Cu or 100 % 72hLC_50_—50 mg/dm^3^ Cu (Som et al. 2009). Sublethal exposure resulted in an increase of abundance of both erythrocyte and leukocyte precursors in head kidney hematopoietic tissue, while under lethal conditions, their number initially decreased, and then increased until the end of the experiment. The authors also observed an increase in blast cell abundance in Cu-exposed fish.

Studies of the effects of metals on hematopoietic tissue of other vertebrates also show their hematotoxic potential. Reduction of hematopoietic potential that resulted in anemia was observed in cadmium-treated rat (Mitsumori et al. 1998). The results of in vitro study revealed that cadmium, lead, and silver adversely affected erythropoiesis in rat bone marrow (Lutton et al. 1984). Van Den Heuvel et al. (1999, 2001) observed that cadmium was more toxic to human and murine hematopoietic progenitor cells than lead, erythroid cell being more sensitive than myeloid precursors. Celik et al. (2005, 2009) reported genotoxic and cytotoxic action of cadmium upon erythroid cells in rat bone marrow.

Almost no data are available on the effects of heavy metals on proliferative and apoptotic activity of fish hematopoietic tissue. The only results were presented by Som et al. (2009) who observed an increase in *Labeo rohita* blast cell apoptosis during both lethal and sublethal Cu exposures, while their proliferation rate increased only under sublethal conditions. These results show concentration-related effect of copper on hematopoietic potential of fish.

There are, however, some data showing the effects of cadmium and copper on proliferative and apoptotic activity in other fish tissues. According to Brunelli et al. (2011), cadmium induced increase in proliferation and apoptosis rates in gill epithelium of *Thalassoma pavo* which was followed by a decrease. Lundebye et al. (1999) reported a significant increase in proliferation and apoptosis rate of intestine epithelial cells of *Salmo salar* fed Cd-containing feed. Activation of both cell proliferation and apoptosis in the intestine of *Liza aurata* from metal-polluted natural environment was also reported by Ferrando et al. (2005). According to Berntssen et al. (2001) and Garcia-Santos et al. (2011), an increase in cell proliferation rate in cadmium-exposed *S. salar* and *Sparus aurata* may be a protective mechanism reducing adverse effect of metal on fish tissues. According to Hernandez et al. (2011), *Danio rerio* larvae subjected to 19.5 mg/dm^3^ of CuSO_4_ for 2 h showed an increase in frequency of apoptosis of various types of cells, head kidney cells being more sensitive than brain neurons and gill epithelial cells. Monteiro et al. (2009) reported an increase in proliferation rate of gill epithelial cells in Cu-exposed *Oreochromis niloticus* which was not accompanied by activation of apoptosis. Massive mitotic activity was detected using PCNA immunostaining in olfactory epithelium of *Tilapia mariae* following 4-day exposure to 20–100 μg/dm^3^ of Cu (Bettini et al. 2006). These observations confirm that cell proliferation is involved in tissue regeneration after metal-induced damage.

Metal-induced activation of cell proliferation and apoptosis was reported also in mammals. According to Habeebu et al. (1998), cadmium induced a dose- and time-dependent activation of murine hepatocyte proliferation and apoptosis. Tsangaris and Tzortzatou-Stathoupoulou (1998) observed that cadmium induced apoptosis of human immune cells, B lymphocytes being more sensitive than T cells and lymphoblasts. Cadmium-induced apoptosis of murine thrombocytes was reported by Fujimaki et al. (2000), and apoptosis of *Anas platyrhynchos* erythrocytes by Romero et al. (2008).

The data obtained in the present study indicate that cadmium and copper may affect hematological and immune status of fish organism by disturbing the process of hematopoiesis. Hematopoietic precursor cells are sensitive to intoxication and heavy metals enhance the rate of their apoptotic destruction. On the other hand, hematopoietic system of carp shows high homeostatic potential and tends to compensate cell loss by activation of mitotic divisions. The results showed that toxic effects of both metals were persistent and hematopoietic activity reduced after short-term exposures to cadmium and copper did not recover in 4-week post-treatment. They also showed that low sublethal concentrations of cadmium, and particularly of copper may significantly disturb hematopoietic processes in fish that do not show any other symptoms of intoxication. In conclusion, anemia and immunosuppression often observed in fish intoxicated with copper and cadmium may result from toxic effect of metals on hematopoietic system. Additionally, cellular parameters of hematopoietic tissue: frequency of blast cells and the rate of proliferation and apoptosis are sensitive indicators of sublethal intoxication.
